# Artificial intelligence in precision medicine: transforming disease subtyping, medical imaging, and pharmacogenomics

**DOI:** 10.1042/ETLS20240011

**Published:** 2025-08-20

**Authors:** Andrea Rodriguez-Martinez, Dilini Kothalawala, Rodrigo M. Carrillo-Larco, Antonios Poulakakis-Daktylidis

**Affiliations:** 1 Medical Research Council Centre for Environment and Health, Department of Epidemiology and Biostatistics, School of Public Health, Imperial College London, London, U.K; 2 BenevolentAI, London, U.K; 3 Hubert Department of Global Health, Rollins School of Public Health, Emory University, GA, U.S.A.

**Keywords:** artificial intelligence, computational biology, drug discovery, precision medicine

## Abstract

Precision medicine marks a transformative shift towards a patient-centric treatment approach, aiming to match ‘the right patients with the right drugs at the right time’. The exponential growth of data from diverse omics modalities, electronic health records, and medical imaging has created unprecedented opportunities for precision medicine. This explosion of data requires advanced processing and analytical tools. At the forefront of this revolution is artificial intelligence (AI), which excels at uncovering hidden patterns within these high-dimensional and complex datasets. AI facilitates the integration and analysis of diverse data types, unlocking unparalleled potential to characterise complex diseases, improve prognosis, and predict treatment response. Despite the enormous potential of AI, challenges related to interpretability, reliability, generalisability, and ethical considerations emerge when translating these tools from research settings into clinical practice.

## Introduction

Since the beginning of the Human Genome Project, there has been exceptional enthusiasm for how genetics and eventually genomics would transform drug discovery. The rapid evolution of genomic technologies has made it possible to comprehensively characterise the genome of both patients and healthy individuals, leading to the identification of hundreds of thousands of genetic variants underlying human disease [1,2]. In addition to genomics, several other high-throughput omics technologies have been developed that are capable of measuring entire pools of transcripts (i.e. transcriptomics), proteins (i.e. proteomics), and metabolites (i.e. metabolomics), as well as epigenetic modifications (i.e. epigenomics) [3,4]. Over the past decade, single-cell omics technologies have revolutionised molecular profiling by providing high-resolution insights into cellular heterogeneity and complexity [5,6]. Importantly, the development of these technologies has been paired with a transition towards integrating molecular data with electronic health records (EHRs), facilitating the identification of relationships between molecular signatures and clinically relevant phenotypes [7–9].

**Figure 1:**
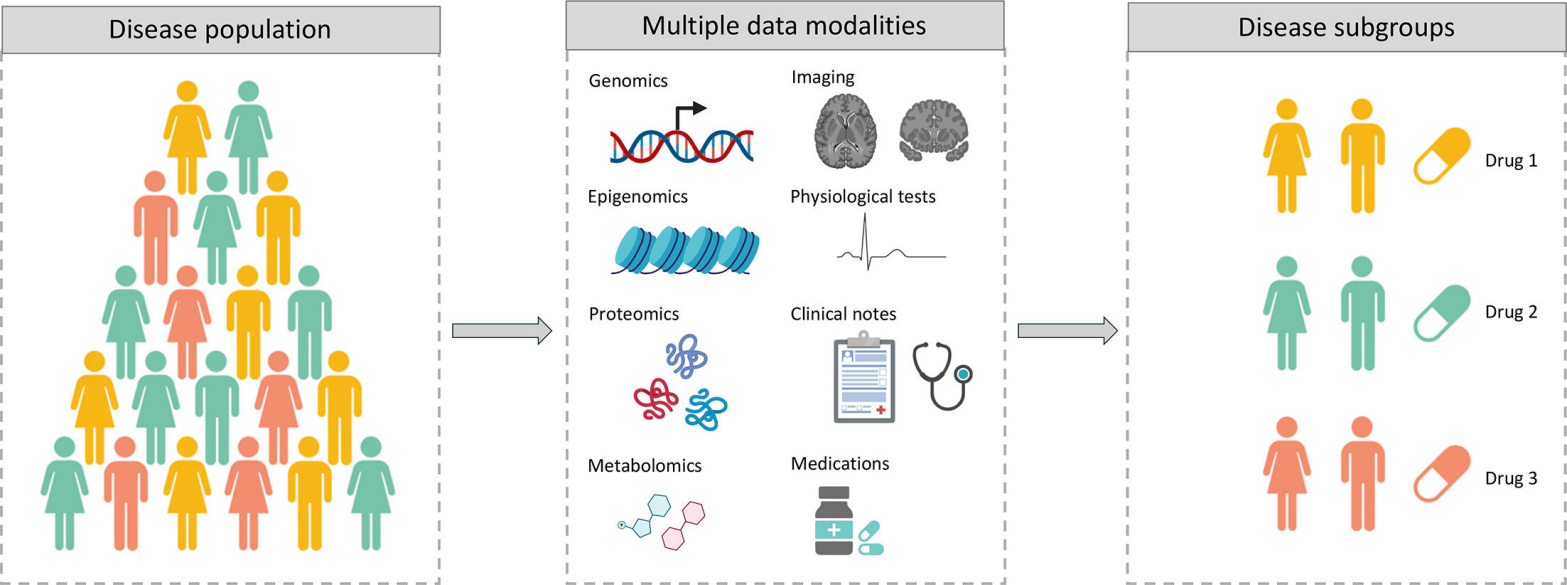
Schematic representation of the precision medicine workflow. This figure was created using BioRender.com.

In this review, we focus on precision medicine as a strategy for patient stratification aimed at designing therapeutic interventions tailored to molecularly defined subgroups [10–12]. Precision medicine leverages molecular data to decipher patterns or signatures characterising specific patient subgroups (Figure 1). These molecular signatures can provide new insights into the mechanisms underlying disease, which ultimately enables the development of drugs that specifically target those mechanisms. For effective clinical translation, it is essential to identify and validate biomarkers that can accurately identify relevant patient subgroups, predict therapeutic response and evaluate treatment effect. In addition, once a patient is matched with the appropriate drug, the treatment regimen must be adjusted according to the patient’s unique characteristics to maximise the therapeutic effect while minimising side effects.

The field of oncology has been the clear pioneer in precision medicine. The transition towards targeted therapies has been motivated by the severe side effects and limited efficacy of conventional treatments, which have only limited selectivity for the malignant cells. Molecular diagnostics and targeted therapies have enabled significant breakthroughs in patient survival for previously ‘incurable’ cancers. One of the earliest examples of success in precision oncology was the FDA approval of trastuzumab in 1998, which revolutionised the treatment of patients with HER2-positive breast cancer [13]. Since then, a growing number of targeted therapies have been developed, ranging from monoclonal antibodies and small molecule drugs that inhibit cell proliferation pathways to targeted immunotherapies [14,15]. In addition to oncology, precision medicine holds an enormous potential to improve patient outcomes for many other therapeutic indications [16–22].

The advent of big data has dramatically enhanced the capabilities of precision medicine. The exponential increase in data generation from different omics modalities, EHRs, and medical imaging has led to a vast and complex information landscape. This surge in data has created the need for advanced data processing and analytical tools. Artificial intelligence (AI) is at the front of this revolution, enabling the detection of hidden patterns from these high-dimensional and complex datasets. AI has shown great potential for characterising complex diseases, prognostication, and predicting treatment response [23–26]. Despite the many opportunities that AI offers, challenges arise when translating these new tools from research settings into clinical practice. These challenges span issues of interpretability, reliability, and ethical considerations [27,28].

In this review, we focus on the advancements of AI in three key precision medicine applications: disease subtyping, medical imaging, and pharmacogenomics. We discuss the disease areas that have seen the most significant advancements in precision medicine and examine the current challenges and the potential future directions of precision medicine in the era of AI.

### Disease subtyping

Precision medicine is often described as an approach that aims to match ‘the right patients to the right drugs at the right time’. A central tenet of precision medicine is disease subtyping, which involves classifying patients into subgroups that share common disease mechanisms. The identification and characterisation of these subgroups, and their corresponding biomarkers, enable the development of tailored treatments, ultimately leading to improved patient outcomes [29]. A growing body of research has explored different disease subtyping methods, ranging from simple models based on clinical observations alone, to complex machine learning (ML) models that integrate multiple omics modalities. Diseases such as cancer, cardiometabolic diseases, neurodegenerative diseases, and infectious diseases have been extensively investigated through the lens of subtyping [30–34].

Traditionally, disease subtyping has been based on the observation of patterns or groups of outlier patients during routine clinical practice. This approach has led to the identification of clinically relevant subgroups across various conditions. For example, in inflammatory bowel disease, patients have been subtyped into Crohn’s disease and ulcerative colitis based on the clinical observations and endoscopic findings. Similarly, asthma patients have been classified into subgroups such as eosinophilic and non-eosinophilic asthma. However, this traditional approach is limited by the ability of clinicians to detect patterns amongst the patients they have encountered, often leading to less comprehensive or nuanced subtyping. In the last two decades, the advent of high-throughput omics technologies and EHRs has revolutionised disease subtyping. These technologies enable a computational- and ML-driven approach that uses large, multi-dimensional datasets to identify common patterns amongst patients at phenotypic, cellular, and molecular levels, leading to the identification of more granular disease subgroups.

**Figure 2:**
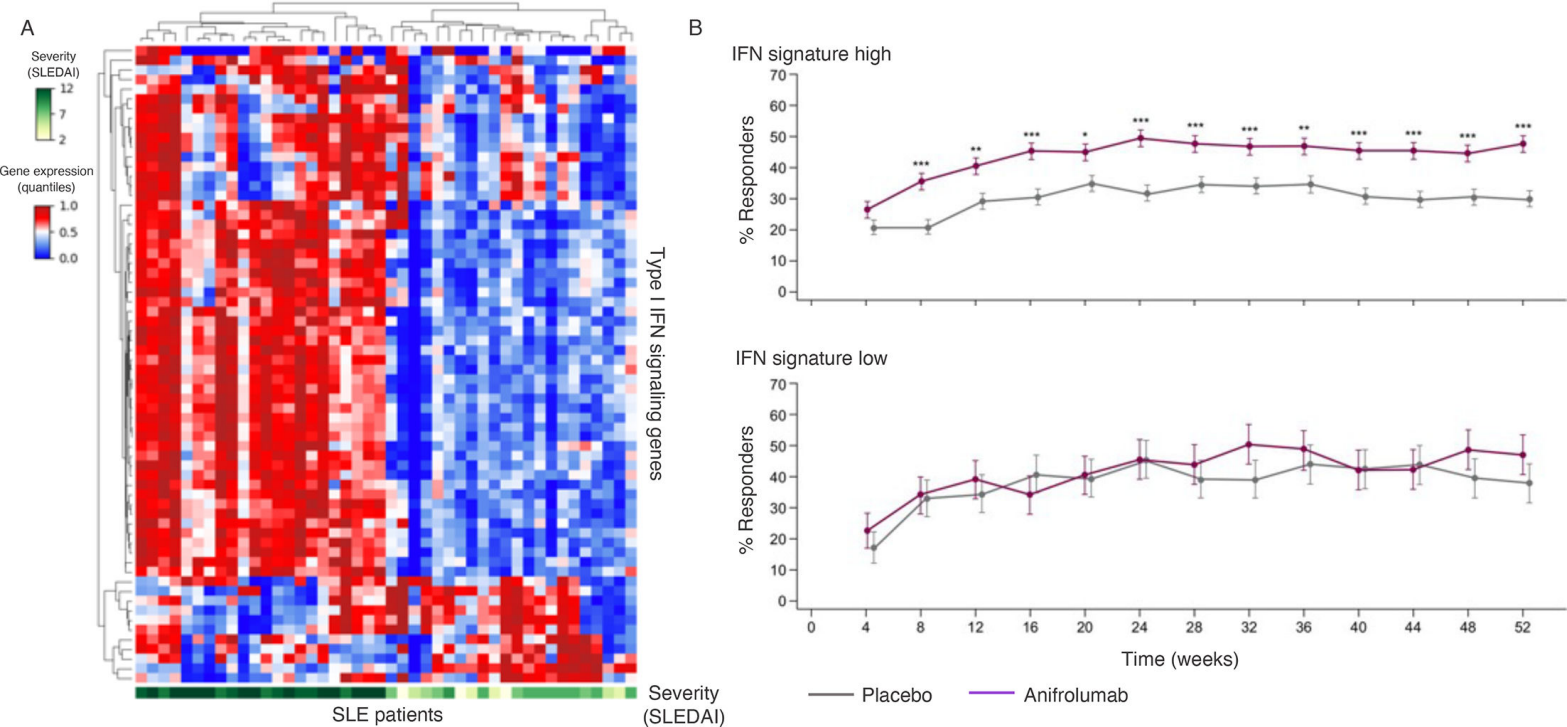
Targeting type 1 interferon (IFN) signalling in systemic lupus erythematosus (SLE). (**A**) Expression profiles of type 1 IFN signalling genes in peripheral blood from 45 SLE patients (GSE228066). Genes and samples are clustered based on hierarchical clustering analysis. The bottom horizontal bar shows the SLE Disease Activity Index (SLEDAI) score for each patient. The heatmap was generated using the PyComplexHeatmap package [41]. (**B**) Response estimates for anifrolumab or placebo arms from 4 to 52 weeks in patients with high or low IFN signature levels at baseline (adapted from [17]).

The overarching goal of computationally driven disease subtyping is to discover clinically relevant patient subgroups from multi-dimensional data. In molecular subtyping, this typically involves applying unsupervised learning techniques to omics data, using methods such as hierarchical clustering analysis and matrix factorisation [35–38]. The resulting subtypes are then characterised based on clinical metadata, to select those associated with clinically relevant characteristics (e.g. survival and disease severity). This approach has been used across a wide range of disease conditions, replicating previously recognised disease subgroups (e.g. subgroups defined based on immunohistochemistry) and uncovering new clinically relevant subgroups. For example, clustering of gene expression patterns from peripheral blood cells from systemic lupus erythematosus (SLE) patients led to the identification of a subgroup of severe lupus patients enriched for interferon (IFN)-inducible genes [39]. This result was replicated in several studies, providing strong biological rationale for the development of SLE therapies that block IFN signalling, and led to the recent approval of anifrolumab [40]. In addition, an ‘IFN gene signature’ was developed to identify the SLE patients who are more likely to benefit from this treatment [17] (Figure 2).

**Figure 3:**
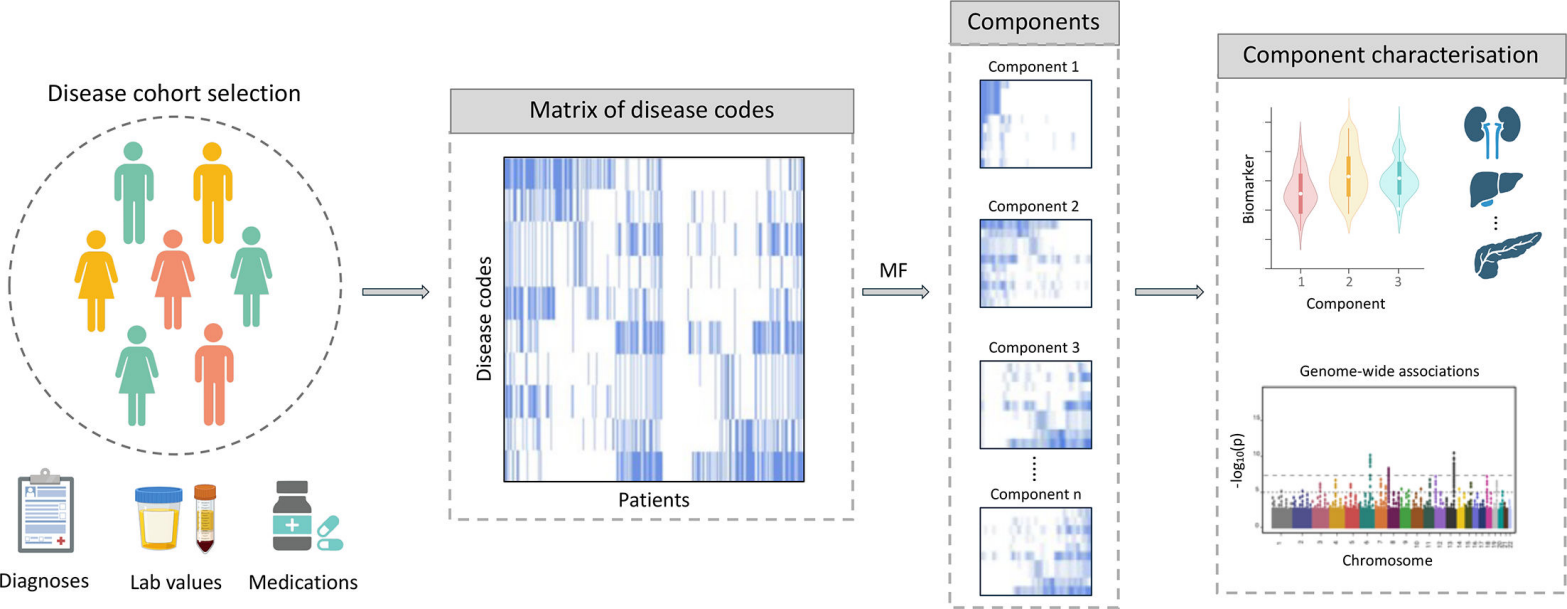
Schematic representation of our pipeline to derive patient subgroups from electronic health records (EHRs). First, a disease cohort is defined based on multiple data sources, including diagnoses, biomarker, and medication data. Next, a matrix representing disease codes (e.g., ICD-10 codes) occurrences across patients is generated. This matrix of disease codes is decomposed into multiple components using matrix factorisation (MF) analysis. The resulting components are further characterised based on biomarker and genetic data, to select those that are clinically and biologically relevant. This figure was created using BioRender.com.

The increasing availability of longitudinal EHRs is driving a new era of disease subtyping, enabling the discovery of novel patient subgroups based on common disease trajectories or comorbidity patterns [42,43]. EHRs consist of heterogeneous structured and unstructured data modalities, including demographic, diagnosis, medication, biomarker, and imaging data. These records provide snapshots of a patient’s health status and have created unprecedented opportunities for patient stratification using ML-driven approaches. Resources that collect both EHR and omics data, such as the UK Biobank [9], the *All of Us* Research Program [44], and *Our Future Health* [45], make it possible to decipher the molecular mechanisms underlying patient subgroups derived from EHRs. For example, we previously developed and validated an end-to-end scalable ML framework to derive patient subgroups from EHRs and further characterise them from a molecular perspective through association analysis with biomarker and genomics data [46]. This approach has been used to characterise patient subgroups across several complex disorders, such as type 2 diabetes [47] (Figure 3). Similarly, Patel et al. [48] described a workflow for identifying coronary artery disease patient subgroups using EHRs and further characterising them through associations with genomics data [48].

Historically, most omics-based disease subtyping studies have used single-data-type study designs. While this approach has enabled the discovery of disease subtypes, analysing a single data type at a time often fails to capture the full complexity of a disease and its molecular phenotypes. Comprehensive subtyping requires integrating multiple data modalities (e.g. transcriptomics, proteomics, and metabolomics). Over the past decade, there has been a considerable increase in studies generating multi-modal datasets, accompanied by the development of novel methods for integrative analysis [49–51]. The simplest methods involve *ad-hoc* combinations of results from independent analyses of different data modalities. More advanced approaches are based on joint analysis of multiple data types. A popular method is similarity network fusion (SNF), which constructs networks of samples (e.g. patients) for each data modality and then efficiently fuses them into a single network that represents the comprehensive underlying data [52]. When tested on five types of cancer, SNF combining mRNA expression, DNA methylation, and miRNA expression outperformed single modality approaches in subtype identification and survival prediction [52]. Despite the availability of various powerful integrative methods, several challenges need to be addressed to realise the full potential of multi-omics. These challenges include differences in sensitivity across omics technologies, the presence of missing values (e.g. a set of omics measurements missing for some samples), the difficulty of interpreting multilayered models, and issues regarding computational performance and scalability [53].

### Medical imaging

Medical imaging is one of the most promising clinical applications of AI. Radiological images are routinely acquired in clinical practice and are a key component for disease diagnosis, guiding treatment, and evaluating response to therapy. Medical imaging is unique in that it captures spatially resolved three-dimensional information about disease in a non-invasive manner. In addition, serial imaging allows for temporal comparison, which is particularly relevant for disease monitoring. In routine practice, radiological image interpretation is based on a qualitative evaluation from a radiologist, along with a few metrics such as lesion diameter or metabolic activity. As such, only a small fraction of the wealth of quantitative information is extracted from medical images in conventional clinical workflows.

Radiomics is an emerging field focused on the transformation of medical images into a mineable high-dimensional feature space [54,55]. This approach presents unique opportunities in precision medicine by enhancing diagnosis, prognostication, and treatment response prediction [56,57]. In drug development, radiomics holds immense potential for discovering novel pharmacodynamic, predictive, and safety biomarkers [58,59]. Radiomics offers several advantages over other omics modalities that rely on invasive biopsies. The latter typically only capture disease at a specific anatomical site, are expensive to acquire, and are often impractical to take at several time points. In contrast, radiomics relies on images that can be obtained at low risk or inconvenience to the patient and that cover the whole extent of the disease considering inter-lesional differences or disease heterogeneity.

As we enter the next era of precision medicine, big data, and AI, the term radiogenomics has emerged to describe the integration of radiomics with other omics molecular modalities [60,61]. One of the key applications of radiogenomics is to predict molecular profiles from image phenotypes in a non-invasive, convenient, and inexpensive manner. Several radiogenomics studies have successfully predicted clinically relevant genetic profiles for breast cancer, lung cancer, pancreatic cancer, and gliomas, from imaging data [62–65]. Other radiogenomics studies have focused on the joint modelling of radiomics and other omics modalities for disease diagnosis, subtyping, and prediction of treatment response. For example, Crispin-Ortuzar et al. developed a promising ML framework to predict response to neoadjuvant chemotherapy in ovarian cancer patients based on the integration of baseline clinical characteristics, molecular biomarkers, and radiomics features [66].

ML has been successfully used for computer vision tasks across multiple fields, including medical imaging classification [67,68]. Historically, supervised learning has been the dominant approach for imaging analysis. The earliest ML methods focused on feature engineering, which involves computing features specified by domain experts before classification. The main disadvantage of these methods is that defining the hand-crafted features requires expert input and it is often a very time-consuming task. Deep learning avoids such feature engineering by autonomously learning the most relevant predictive features from images [69]. Convolutional neural networks (CNNs) are the most popular deep learning method for pattern recognition tasks in medical images [70,71]. CNN-based architectures have shown to match, or even surpass, human experts in tasks, such as identifying diabetic retinopathy in retinal images, detecting malignant lesions from mammograms, classifying skin lesions or predicting EGFR status in lung adenocarcinoma using CT scans [72–75]. Nevertheless, a major challenge in supervised learning tasks, particularly in deep learning, is that they require large amounts of expert-labelled datasets for model training. Additionally, these models are often focused on highly specific tasks and might struggle to generalise to new problems. Recently, self-supervised learning has emerged as a promising solution to overcome these obstacles [76,77].

From diagnostics to prediction of treatment response, new applications for radiomics are rapidly being developed. However, several clinical and technical challenges hinder the implementation of radiomics in routine clinical practice and drug development programmes [58,60,78]. One of the largest hurdles for radiomics is the lack of reproducibility of results across studies, often due to the relatively small sample sizes and differences in protocols for image acquisition, processing, and modelling. To identify truly reproducible radiomics signatures, large and heterogeneous patient cohorts must be collected from multiple centres. In addition, the data should be analysed using advanced methods capable of addressing data heterogeneity.

### Pharmacogenomics

Pharmacogenomics, the use of genomic information to predict drug response, is arguably the earliest application of precision medicine. Genetic variation accounts for a considerable proportion of inter-individual variability in drug response. Genetic variants can influence a drug’s pharmacokinetics (e.g. absorption, distribution, metabolism, and elimination) or its pharmacodynamics (e.g. by modifying the interaction with its target) [79,80]. Pharmacogenomics can help stratify patients into responders and non-responders, avoid side effects, and optimise drug dose. For instance, inter-individual variability in response to warfarin, the most commonly used oral anticoagulant, is largely due to polymorphisms in two enzymes: CYP2C9, which is involved in warfarin metabolism, and VKORC1, the pharmacological target of warfarin [81]. This has led to the development of pharmacogenomics algorithms to optimise warfarin dosing and the revision of its drug label to recommend genetic testing for dose selection [81,82].

Over the past few decades, numerous studies have reported links between genetic variants and drug response, leading to the inclusion of pharmacogenomics information on the labels of hundreds of therapeutic products [79,83]. The clinical implementation of pharmacogenomics has made important strides in oncology, where treatments often target specific mutations in the tumour genome. However, its application in other therapeutic areas has not progressed as rapidly. Several factors have contributed to this lag in clinical implementation [83,84]. A major issue is the low statistical power and limited reliability of many pharmacogenomics studies, often due to small sample sizes relative to the frequencies of the genetic variants under study, small effect sizes of most individual genetic variants, inaccurate cohort definitions, and lack of validation of results in independent cohorts. The resulting uncertainty in the identified gene–drug relationships is reflected in the limited consensus between pharmacogenomics labels as considered by different regulatory agencies [85]. Another key bottleneck is the lack of functional characterisation of many of the genetic variants identified in pharmacogenomics studies [84].

The gap between the identification and use of genetic variants for precision medicine applications is expected to diminish in the future. The increasing availability of population-scale resources that integrate EHRs with genomics offers unique opportunities for identifying robust pharmacogenomics associations [86,87]. In addition, recently developed AI models hold enormous potential for characterising both coding and non-coding genetic variants associated with drug response [88,89]. Furthermore, polygenic risk scores, which combine the effects of many genetic signals across the genome, have emerged as promising tools for improving patient stratification and drug response prediction compared with individual genetic variants [90–92]. These technological advancements and comprehensive data generation efforts are very likely to drive the field forward. However, as we describe in further detail in the outlook section, realising the full potential of pharmacogenomics in clinical practice requires overcoming several logistical, ethical, and regulatory challenges.

### Outlook

The integration of multi-omics data, EHRs, and AI into the sphere of precision medicine is revolutionising drug development. Important progress has been made in disease subtyping, medical imaging, and pharmacogenomics, with oncology leading the way in clinical implementation. A key factor driving progress in oncology is the greater availability of patient-specific data, particularly tissue samples obtained through biopsies, which offer critical insights into tumour biology. In contrast, other fields—such as neurology and cardiometabolic diseases—primarily depend on molecular profiles from blood or urine samples, which may provide a less comprehensive view of the disease. Advancements in non-invasive tissue profiling techniques, such as radiomics, hold promise for bridging this gap and enhancing disease characterisation across disease areas.

Looking ahead, several key factors are set to drive progress and expand the scope of precision medicine. The increasing availability of population-scale resources that integrate omics data and EHRs, coupled with rapid analytical and computational advancements, is paving the way for a new era of data-driven precision medicine. Large language models hold immense promise in this space. These innovative generative AI technologies excel at processing, synthesising, and integrating information from diverse data modalities. Their capacity to scalably train on vast and varied multi-modal datasets through self-supervised learning enables broad applicability to a wide range of downstream problems.

In the future, it may be possible to envision an ideal healthcare system, where patients undergo extensive molecular and imaging testing, with all data seamlessly integrated through advanced AI algorithms to support personalised treatment and prevention strategies. Patient data will continually feed back into the AI models, refining and enhancing their predictive power. This iterative learning process will deepen our understanding of diseases, uncover new therapeutic targets, and drive the development of tailored therapies that are more effective and have fewer side effects.

However, realising this vision of AI-driven precision medicine requires several key challenges to be addressed. First, the integration of AI in healthcare raises significant ethical considerations related to patient privacy, informed consent, and the responsible use of AI insights. To address these concerns, it is vital to develop strong frameworks that safeguard patient rights and ensure transparent data usage. Strategies like federated learning, synthetic data, and embedding representations can facilitate data sharing while minimising breaches. Second, many AI models function as ‘black boxes’, which can hinder trust amongst scientists and clinicians. Improving the explainability and interpretability of these models is essential for their clinical adoption [93,94]. Approaches like the one described by Patel et al. [95] represent a promising solution to derive faithful and quantitative explanations from language model predictions [95]. Lastly, ensuring AI models are accurate and reliable across diverse populations and clinical settings is crucial. Collaborating on large-scale projects to access varied and representative datasets, as well as making the models open source, can enhance model validation in different populations. Clear guidelines for conducting experiments and reporting metrics are also necessary to ensure robust and credible performance claims.

Beyond AI-specific challenges, broader logistical, ethical, and regulatory challenges need to be addressed for the seamless integration of precision medicine into healthcare systems. Countries and regulatory bodies need to establish clear guidelines on data collection, storage, access, security, and patient consent. Another critical challenge is the availability of resources. This encompasses logistical factors, such as laboratory infrastructure, access to necessary tests and devices, and the availability of trained personnel. Skilled professionals are essential not only for analysing omics data but also for interpreting results and translating them into clinically actionable insights. Cost and financial responsibility present additional hurdles. A fundamental question remains: who will bear the costs of omics testing and its associated components? The answer may vary by country and healthcare system. It could fall on private healthcare providers with greater financial resources, new industry players offering omics services, or public health systems that assume these costs as part of broader health initiatives. Finally, the diversity of available and legacy systems presents a challenge in seamless integration of records and data, especially in the presence of multiple EHR systems. This diversity should be solved to maximise the available data, leading to the development of more applications and enhancing the generalisability of findings.

Addressing these challenges will require collaborative efforts from researchers, clinicians, policymakers, regulatory agencies, healthcare providers, and industry stakeholders. By fostering an environment of innovation and responsible AI development, we can pave the way for a future, where precision medicine is a standard of care for all patients.

**Summary**

- Precision medicine aims to match ‘the right patients with the right drugs and the right time’.
- The exponential growth of omics data and electronic health records, coupled with the development of advanced computational- and AI-driven methods, is revolutionising precision medicine.
- Despite the significant potential of AI tools, several challenges arise when transitioning these tools from research into clinical practice.

## Abbreviations

AI: artificial intelligence
CNNs: convolutional neural networks
CT: computed tomography
EHRs: electronic health records
IFN: interferon
ML: machine learning
SLE: systemic lupus erythematosus
SNF: similarity network fusion
